# Ubiquitin-driven regulation of immune checkpoints in lung cancer: Mechanisms and therapeutic implications (Review)

**DOI:** 10.3892/etm.2026.13243

**Published:** 2026-07-15

**Authors:** Lin Chai, Lin-Rong Pang, Yi-Ting Li, Jia-Hui Wang, Jun Chen, Zu-Guo Yuan, Xiao-Feng Jin

**Affiliations:** 1 Department of Chemoradiotherapy, The Affiliated People's Hospital of Ningbo University, Ningbo, Zhejiang 315040, P.R. China; 2 Zhejiang Key Laboratory of Pathophysiology, Department of Biochemistry and Molecular Biology, Health Science Center, Ningbo University, Ningbo, Zhejiang 315211, P.R. China

**Keywords:** ubiquitination, deubiquitination, immune checkpoints, lung cancer, therapy

## Abstract

The ubiquitin-proteasome system is a master regulator of anti-tumor immunity in lung cancer, which primarily functions through controlling the stability of immune checkpoint proteins. The present review offers a synthesis concerning how a dynamic balance between E3 ubiquitin ligases (E3s) and deubiquitinases (DUBs) dictates the fate of key checkpoint proteins, including programmed cell death protein 1/programmed death-ligand 1, lymphocyte-activating gene 3 and B7 homolog 4. Although specific E3s are known to promote checkpoint degradation to enhance T-cell function in certain contexts, and DUBs frequently stabilize these proteins to foster immune evasion, these effects are context-dependent; for example, certain E3s are paradoxically able to promote immune evasion, whereas the inhibition of select DUBs synergizes with immune checkpoint blockade. This regulatory interplay extends to core oncogenic pathways, including the phosphoinositide 3-kinase/AKT and mitogen-activated protein kinase signaling pathways, which indirectly modulate checkpoint expression. Therapeutically, targeting these enzymes with various agents, such as the ubiquitin-specific peptidase 7 inhibitor P5091 or the repurposed drug canagliflozin, has the effect of synergizing with immune checkpoint blockade through reshaping the tumor microenvironment. However, clinical translation is challenged by tumor heterogeneity, pathway redundancy and the complexity of the ubiquitin network. Future progress in this area hinges on precision drug design, predictive biomarker development and rational combination therapies that are informed by a deeper mechanistic understanding of ubiquitin-driven immune regulation.

## 1. Introduction

Lung cancer is a leading global health burden, accounting for a marked proportion of cancer-associated deaths. Histologically, this malignancy is classified into two distinct subtypes: Non-small cell lung carcinoma (NSCLC) and small cell lung carcinoma (SCLC). NSCLC accounts for ~85% of all cases, whereas SCLC constitutes ~15% of pulmonary malignancies. Lung cancer is characterized by its aggressive biological behavior, rapid proliferation kinetics and early systemic dissemination (1). Smoking, occupational exposure, environmental pollution and family history are all risk factors for lung cancer. Although numerous treatments for lung cancer are available, including radiotherapy, surgery and chemotherapy, the prognosis for the disease remains poor (2). The NSCLC and SCLC types of metastatic lung cancer are both associated with poor survival, with a 5-year survival rate of ~4% (3).

With the discovery of immunotherapy, however, the treatment landscape for advanced lung cancer has fundamentally changed. Immunotherapy for lung cancer primarily involves immune checkpoint blockade. Tumor cells are able to evade the surveillance and attack of the immune system using immune checkpoint-associated mechanisms. Immune checkpoint inhibitors (ICIs) serve to restore the antitumor activity of T cells by blocking these inhibitory signals, and compared with traditional chemotherapy drugs, ICIs have been shown to improve overall survival rates and cause fewer adverse reactions (4).

Immune checkpoints comprise a network of inhibitory receptors and ligands that physiologically maintain self-tolerance and prevent excessive immune damage. Key members include cytotoxic T-lymphocyte-associated protein 4 (CTLA-4) (5), programmed cell death protein 1 (PD-1) (6), programmed death-ligand 1 (PD-L1) (7), lymphocyte-activating gene 3 (LAG-3) (8), B7 homolog 4 (B7-H4) (9) and emerging targets such as T-cell immunoglobulin and mucin-domain containing-3 (TIM-3) (10) and T-cell immunoreceptor with Ig and ITIM domains (TIGIT) (11). Under physiological conditions, engagement of these molecules suppresses T-cell activation and preserves immune homeostasis. By contrast, tumors exploit these pathways to establish an immunosuppressive microenvironment. For example, in tumor cells, PD-L1 expression is frequently upregulated in response to interferon-gamma (IFN-γ) stimulation within the tumor microenvironment (12). This leads to T-cell dysfunction, encompassing diminished activation, impaired proliferation, reduced cytokine secretion (including interleukin-2 and interferon-γ) and attenuated cytotoxicity (13), subsequently allowing sustained tumor proliferation. Consequently, the advent of ICIs that disrupt these interactions has transformed lung cancer therapy. However, one disadvantage is that the clinical efficacy of ICIs is limited by primary and acquired resistance (14). Emerging evidence indicates that post-translational modifications (PTMs), especially ubiquitination, notably govern the stability and function of immune checkpoint proteins (15). Although TIM-3 and TIGIT hold considerable clinical promise as next-generation immune checkpoint targets, with multiple inhibitors (such as sabatolimab, tiragolumab and ociperlimab) currently undergoing phase I-III clinical trials for NSCLC and other solid tumors (16), their direct regulation by ubiquitin-modifying enzymes in lung cancer remains to be fully elucidated. While the exact E3 ligase responsible for TIM-3 proteasomal degradation has not yet been identified, the SCF ubiquitin ligase complex is known to regulate the intracellular transport of phosphatidylserine, a key TIM-3 ligand (17). By contrast, TIGIT has been demonstrated to undergo K48-linked polyubiquitination mediated by the E3 ligase STUB1, particularly under hypoxic conditions, leading to its proteasomal degradation (15).

**Figure 1:**
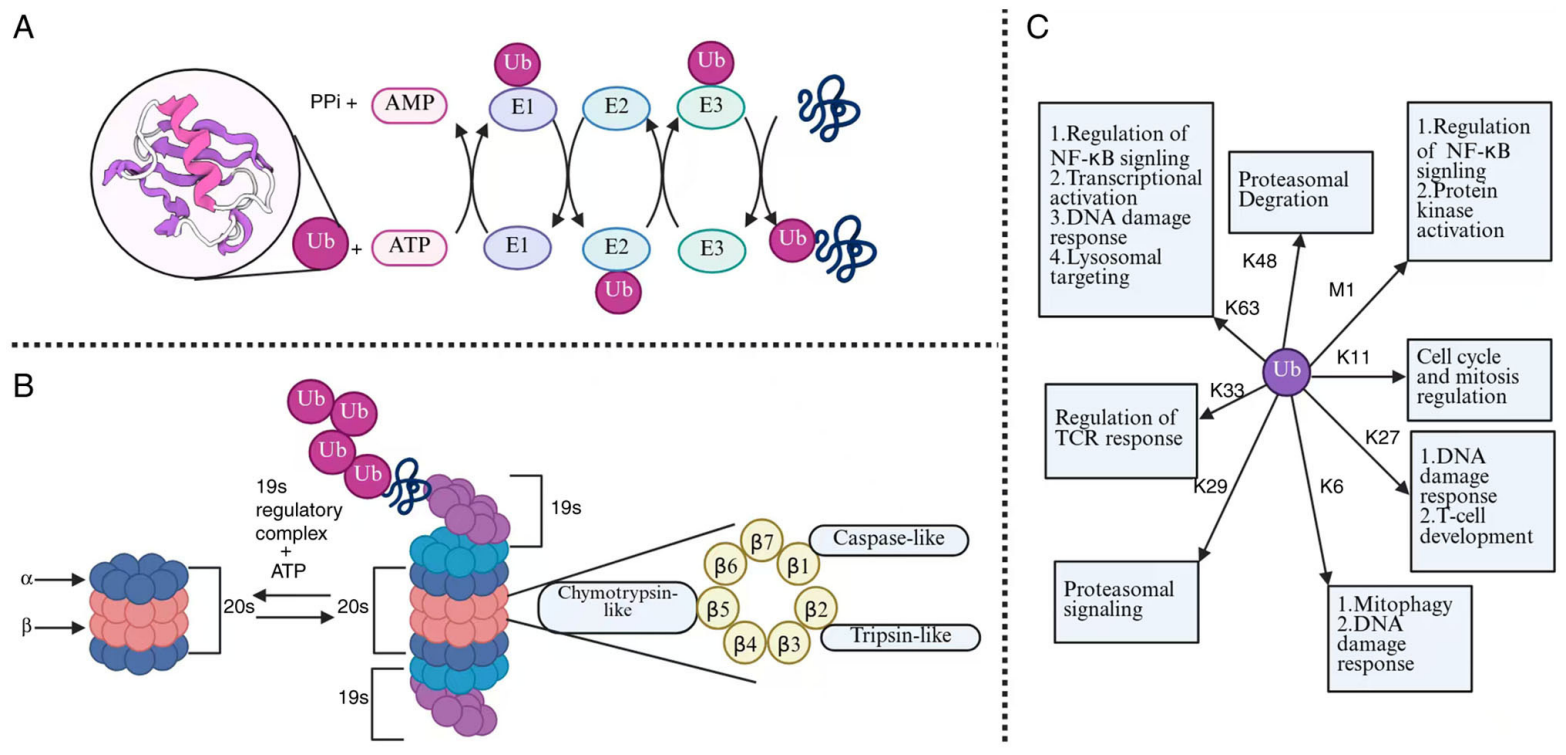
Ubiquitination modification process, the ubiquitin-proteasome pathway and the types of ubiquitination modifications. (A) The ubiquitination process is depicted. First, with ATP providing energy, the ubiquitin-activating enzyme E1 activates the ubiquitin molecule. Secondly, the ubiquitin-activating enzyme E1 transfers the activated ubiquitin molecule to the ubiquitin-binding enzyme E2. Finally, the ubiquitin ligase E3 attaches the bound ubiquitin to the target protein. (B) The ubiquitin-proteasome pathway is shown. Ubiquitinated proteins bind to the 19S complex and are degraded at the proteolytic β subunit. The 19S subunit binds to multiple ubiquitin chains, and ATP unfolds the protein substrate and transfers it to the 20S core particle. The protein is subsequently degraded into small oligopeptides <25 amino acids in length through the 20S core. This mediates ubiquitin-independent protein degradation. (C) The types of ubiquitination modifications. In the ubiquitin chain, the ubiquitin portion can bind to its lysine residues (K11, K27, K6, K29, K33, K63 and K48) or the N-terminal methionine residue (M1). Each chain is recognized by different ubiquitin binding domains, thereby targeting proteins in specific signaling pathways. Ub, ubiquitin.

The ubiquitin-proteasome system (UPS) is a complex protein degradation system composed of the ubiquitin system and proteasomes. The proteasome is mainly involved with the degradation mechanism, comprising the 19S regulatory particle, the 20S core particle and the 26S proteasome (18). It recognizes and degrades proteins with ubiquitin chains. The UPS also includes components such as ubiquitin, E1 ubiquitin-activating enzyme, E2 ubiquitin-conjugating enzyme and E3 ubiquitin-ligase. These components serve to label target proteins for degradation, and through a succession of enzymatic cascades involving the E1, E2 and E3 enzymes, ubiquitin is transferred to the lysine residue of the substrate via its C-terminal glycine and ε-amino groups (19). By contrast, deubiquitinating enzymes (DUBs) catalyze the removal of ubiquitin from the substrate. In terms of the underlying mechanism, ubiquitin is first conjugated to E1 ubiquitin-activating enzymes. It is subsequently moved to E2 ubiquitin-conjugating enzymes in an ATP-dependent manner, and then ubiquitin is translocated from the ubiquitin-E2 complex to the lysine residues on the substrate. This process can occur directly via E2 enzymes [aided by RING-type E3 ligases (E3s)], or indirectly through homologous to the E6-AP carboxyl terminus (HECT)- or RING-between-RING (RBR)-type E3s, which accept ubiquitin before transferring it to the substrate (20). The process of ubiquitination is characterized by the action of an E3 ligase, whether RING-type, HECT-type or RBR-type, which determines its specificity (21). The E3 enzyme recognizes and selectively binds specific substrate proteins. Ubiquitins contain seven lysine residues (K6, K11, K27, K29, K33, K48 and K63) that enable the formation of diverse polyubiquitin chains, and these dictate the functional outcomes of target proteins (22). For example, K63-linked chains are essential for signal transduction and DNA damage repair, whereas K48- and K11-linked chains predominantly target proteasomal degradation substrates (23). Ubiquitin ligases (E3s), which mediate these modifications, are classified into five major families: RING E3s, HECT E3s, RBR E3s, cullin-RING ligases and U-box E3s (21). On the other hand, DUBs reverse ubiquitination by cleaving the ubiquitin chains. DUBs are categorized into six distinct subfamilies based on their catalytic domain structure and mechanistic features: Ubiquitin-specific proteases, ubiquitin C-terminal hydrolases, Machado-Joseph disease proteases, ovarian tumor proteases (OTUs), MINDYs and JAMMs (24) (Fig. 1).

## 2. Roles of ubiquitination and deubiquitination in immune checkpoints in lung cancer

As notable regulatory mechanisms, ubiquitination and deubiquitination perform pivotal roles in lung cancer through modulating tumor progression and therapeutic efficacy (25). As aforementioned, the enzymes governing these processes have emerged as promising therapeutic targets, especially through their ability to regulate the stability and function of key immune checkpoints, including PD-1/PD-L1, LAG-3 and CTLA-4. The present review summarizes the functions of ubiquitinating enzymes and DUBs in lung cancer and delves into the underlying mechanisms via which they control immune checkpoint expression and activity (Table I).

### PD-1/PD-L1: A notable immune checkpoint in lung cancer

PD-1 (or CD279) is a 55-kDa transmembrane protein composed of 288 amino acids. Its structure includes an extracellular N-terminal IgV-like domain, a transmembrane domain and a cytoplasmic tail (26). The cytoplasmic tail contains two tyrosine-based motifs located at the N- and C-terminal regions (27). Its ligand, PD-L1 (also known as CD274 or B7-H1), is a member of the B7 family, which functions as a 33-kDa type I transmembrane glycoprotein. The extracellular region of PD-L1 consists of 290 amino acids, featuring both IgV and IgC structural domains (28). Under normal physiological conditions, PD-L1 is expressed by macrophages, various activated T- and B-cells, dendritic cells and certain epithelial cells, especially in response to inflammatory conditions (29).

The PD-1 pathway functions as a key negative feedback mechanism in normal immunity. T-cell activation, triggered by the engagement of T-cell receptors (TCRs) with antigen-major histocompatibility complex (MHC) and costimulatory signals, leads to the upregulation of PD-1 on the T-cell surface. Binding of PD-1 by PD-L1 from antigen-presenting cells subsequently transmits inhibitory signals that suppress T-cell activation, curb cell proliferation and diminish cytokine release, thereby contributing to the maintenance of immune tolerance (30). In the context of tumor immune escape, cancer cells exploit this regulatory mechanism through upregulating PD-L1 expression by means of various mechanisms, such as in response to IFN-γ within the tumor microenvironment (31). Tumor-surface PD-L1 engages PD-1 on tumor-infiltrating T cells, delivering inhibitory signals that lead to both T-cell dysfunction and an impairment of the immune-mediated clearance of tumor cells (32). Consequently, the suppression of T-cell activity both enables sustained tumor proliferation and promotes tumor disease progression.

The activity of the PD-1/PD-L1 pathway is regulated through multiple layers of control, including gene transcription, post-transcriptional modifications, PTMs and exosomal transport (33). Among these, PTMs, such as glycosylation, phosphorylation, ubiquitination, palmitoylation, SUMOylation and acetylation, fulfill particularly notable roles in modulating the stability and protein-protein interactions of PD-1 and PD-L1(34). Notably, substantial evidence over the past decade has established that the expression levels of PD-1 and PD-L1 are markedly influenced by ubiquitin-mediated proteasomal degradation. As a key regulatory mechanism, ubiquitination is essential for controlling diverse cellular processes, including protein stability, receptor internalization and immune responses (35).

**Figure 2:**
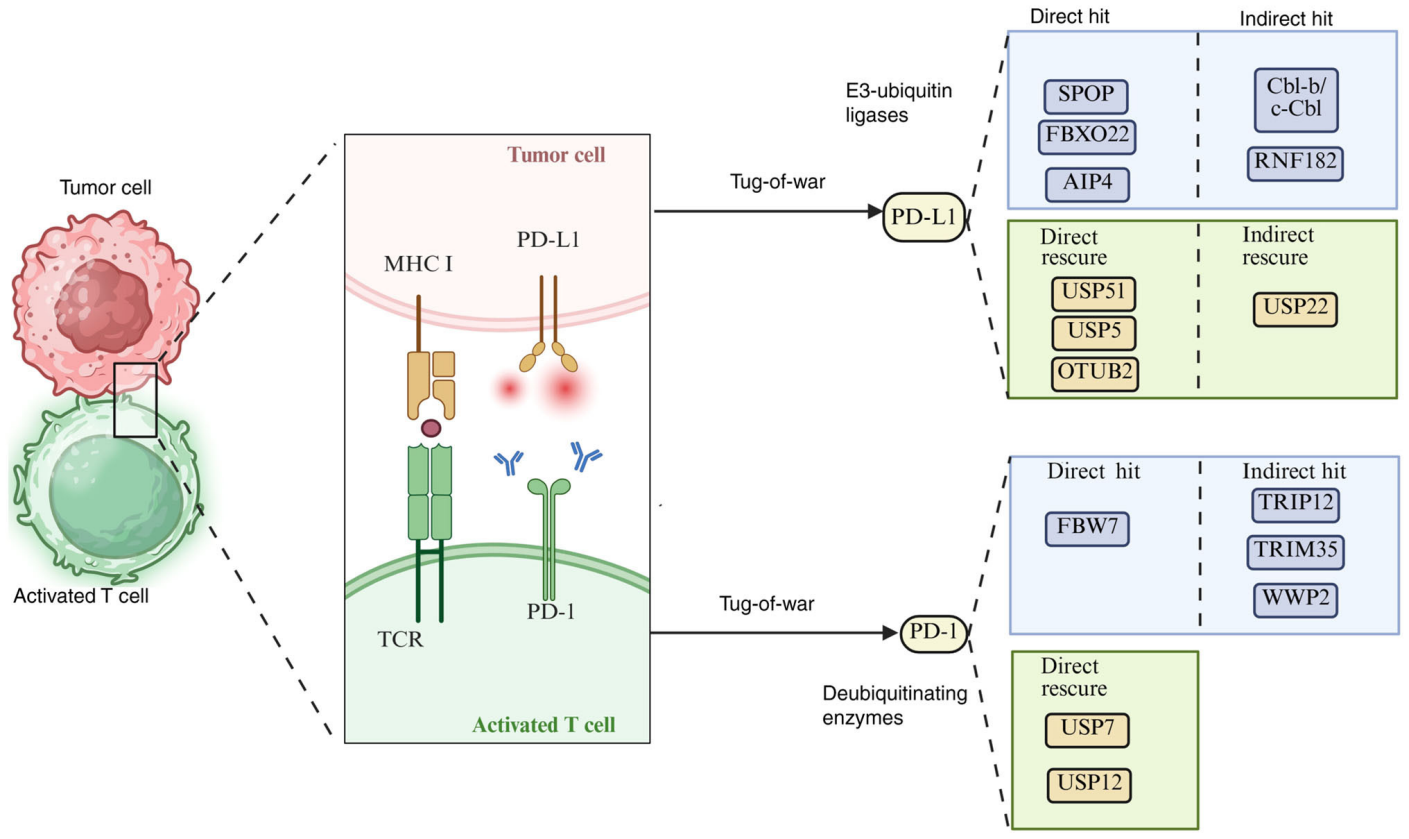
Role of Ubiquitinating and Deubiquitinating enzymes in PD-1/PD-L1 immune checkpoints. A schematic diagram of the ubiquitination regulatory network of the T cell immune checkpoint protein PD-1 and its ligand PD-L1 in the tumor microenvironment, showing the regulatory effects of deubiquitinating enzymes and E3 ligases on the stability and function of PD-1, and elucidating the molecular mechanism via which ubiquitination modification mediates T cell exhaustion and immune escape. PD-1, programmed cell death protein 1; PD-L1, programmed death-ligand 1; MHC, major histocompatibility complex; TCR, T-cell receptor; SPOP, speckle-type POZ protein; FBXO22, F-box protein 22; FBW7, F-box and WD repeat domain-containing 7; AIP4, atrophin-1 interacting protein 4; RNF182, ring finger protein 182; Cbl, Casitas B-lineage lymphoma; USP, ubiquitin-specific peptidase; OTUB2, OTU and ubiquitin aldehyde-binding 2; TRIM35, tripartite motif-containing 35; TRIP12, thyroid hormone receptor interactor 12; WWP2, WW domain-containing E3 ubiquitin-protein ligase 2.

The subsequent part of the present review delineates the sophisticated roles of E3s and DUBs in dynamically shaping the tumor immune microenvironment (TIME). It details a molecular ‘tug-of-war’, wherein specific E3s [for example, speckle-type POZ protein (SPOP), F-box protein (FBXO)22 and F-box and WD repeat domain containing 7 (FBW7)] target PD-L1 and PD-1 for degradation to potentiate anti-tumor immunity. By contrast, counteracting DUBs [for example, ubiquitin-specific peptidase 22 (USP22), ubiquitin-specific peptidase 51 (USP51) and the DUB OTU and ubiquitin aldehyde binding 2 (OTUB2)] stabilize these checkpoints to foster immune evasion; however, these functions are context-dependent. The present review explores how this ubiquitin-mediated regulation extends beyond the checkpoints themselves to orchestrate key oncogenic signaling pathways [for example, the phosphoinositide 3-kinase (PI3K/AKT) and mitogen-activated protein kinase (MAPK) signaling pathways] and modulate broader immune responses, including phagocytic clearance mediated via cluster of differentiation (CD)47. Through highlighting context-dependent regulators such as ubiquitin-specific peptidase 7 (USP7) and ubiquitin-specific peptidase 12 (USP12), the discussion underscores the therapeutic potential of strategically manipulating this equilibrium, which can be achieved either through augmenting E3 activity or by inhibiting specific DUBs, thereby recalibrating the TIME and overcoming resistance to immunotherapy in lung cancer (Fig. 2). Notably, although TIM-3 and TIGIT represent clinically relevant immune checkpoints in lung cancer, their direct regulation by ubiquitin-modifying enzymes remains largely unexplored.

### E3s and DUBs in direct PD-L1 modulation

Beyond simple protein degradation, the ubiquitin system has emerged as a sophisticated signaling network that dynamically shapes the lung cancer immune landscape. E3s function as molecular switches, the activity of which directly determines tumor immune recognition and therapeutic susceptibility (36); however, their effects are not uniformly pro-immunity. SPOP exemplifies how existing drugs can be repurposed to manipulate this system. The ability of canagliflozin to disrupt the sodium-glucose cotransporter-2 (SGLT2)-PD-L1 axis redirects the activity of SPOP toward PD-L1 degradation, thereby creating a window for enhanced T-cell attack (37). Similarly, FBXO22 operates at the intersection of DNA damage response and immunotherapy. The inhibition of cyclin-dependent kinase 5 was unexpectedly shown to strengthen antitumor immunity through FBXO22-mediated PD-L1 control (38). This system demonstrates marked versatility in terms of its regulatory strategies. Although atrophin-1 interacting protein 4 (AIP4) has been shown to collaborate with metastasis suppressor 1 (MTSS1) to channel PD-L1 toward lysosomal degradation (39), ring finger protein 182 (RNF182) and casitas B-lineage lymphoma (Cbl) proteins employ indirect mechanisms. RNF182 dismantles the transcriptional activator p65, whereas Cbl proteins serve to constrain the upstream signal transducer and activator of transcription 3 (STAT3)/AKT/extracellular signal-regulated kinase (ERK) signaling pathway. These diverse approaches converge on the same goal, namely modulating the presence of PD-L1 at the immune synapse.

Not all E3s, however, are involved in pushing toward immune activation. DCUN1D1 (defective in cullin neddylation 1 domain containing 1), also known as SCCRO, functions as an essential co-E3 component of the neddylation E3 complex that promotes cullin neddylation and subsequent CRL activation (40). This E3 enhances PD-L1 expression through activation of the focal adhesion kinase (FAK) signaling pathway, thereby highlighting the delicate balance within this network (41). The collective activity of these competing forces ultimately determines whether the TIME permits or prevents effective immune surveillance, thereby positioning the ubiquitin system as a central arbitrator of immunotherapeutic outcomes in lung cancer.

Beyond transcriptional control, PD-L1 expression is dynamically shaped by DUBs that stabilize PD-L1 through distinct PTMs, thereby creating a notable barrier to effective immunotherapy. Multiple DUBs converge on PD-L1 protection through specialized mechanisms. USP51 directly stabilizes PD-L1 by removing ubiquitin chains at K280/K281, which has the effect of driving dual resistance to both chemotherapy and immunotherapy (42). By contrast, USP22 employs a dual-layer stabilization mechanism: Not only does it deubiquitinate PD-L1 at multiple lysine residues, but it also stabilizes constitutive photomorphogenesis 9 signalosome subunit 5 (CSN5). CSN5 itself functions as a JAMM-family metalloprotease deubiquitinase that directly binds the C-terminal region of PD-L1 and cleaves its ubiquitin moieties via MPN-domain enzymatic activity, thereby preventing proteasomal degradation. Consequently, USP22 fortifies PD-L1 stability through two convergent routes, direct deubiquitination of PD-L1 and indirect stabilization of its upstream guardian CSN5, creating a reinforced USP22-CSN5-PD-L1 signaling circuit that amplifies immune evasion (43,44). OTUB2 operates as an early checkpoint protein that intercepts PD-L1 in the endoplasmic reticulum (ER) to subvert ER-associated degradation, which thereby ensures that it is trafficked to the cell surface (45).

The regulatory landscape has been shown to extend beyond direct protein stabilization. Ataxin-3, a protein encoded by the human *ATXN3* gene that primarily functions as DUB, determines PD-L1 upregulation through transcriptional control. It promotes PD-L1 upregulation through two distinct microenvironmental cues. Upon IFN-γ stimulation, it deubiquitinates and stabilizes both interferon regulatory factor 1 (IRF1) and signal transducer and activator of transcription 3 (STAT3), thereby preserving their ability to bind the PD-L1 promoter and enhance its transcription. Under hypoxic conditions, ATXN3 functions as a deubiquitinase for hypoxia-inducible factor-2α (HIF-2α), preventing its proteasomal degradation; the accumulated HIF-2α subsequently translocates to the nucleus and drives PD-L1 transcription through hypoxia-response elements in the PD-L1 promoter. These convergent transcriptional programs ultimately increase PD-L1 abundance and foster immune evasion (46). Similarly, ubiquitin C-terminal hydrolase L1 was shown to enhance PD-L1 expression through AKT-P65 pathway activation, which creates an immunosuppressive microenvironment that dampens T-cell efficacy (47). Notably, USP5 exemplifies how DUB function is integrated with broader cellular systems. Classically known as isopeptidase T (IsoT), USP5 specifically disassembles unanchored polyubiquitin chains, generated during proteasomal processing or aberrant E2/E3 activity, into monoubiquitin units for recycling. When these free chains accumulate, they competitively bind proteasomal ubiquitin receptors (such as S5a/Rpn10), obstructing the recognition and degradation of authentic ubiquitinated substrates and thereby disrupting global proteostasis. By clearing this inhibitory pool, USP5 preserves proteasome capacity and maintains the cellular monoubiquitin reservoir, which indirectly sustains PD-L1 stability and links the general ubiquitin-proteasome system to specific immune checkpoint control. Beyond proteostasis, USP5 also participates in DNA double-strand break repair, Wnt/β-catenin signaling, and NF-κB-mediated inflammatory responses, further coupling immune checkpoint regulation to genome integrity and oncogenic signaling networks (48,49).

This intricate network reveals how DUBs function as sophisticated modulators of tumor immunity. The diverse range of mechanisms that they employ, ranging from direct deubiquitination to transcriptional reprogramming and pathway activation, demonstrate their potential as therapeutic targets. Nevertheless, the impact of DUB inhibition needs to be evaluated in a context-specific manner, as the net effects elicited depend on the particular substrate and cellular environment.

### E3s and DUBs in PD-1 regulation and contextual immune modulation

Multiple E3s form a complex regulatory network that both modulates the TIME and responses to anti-PD-1/PD-1 immunotherapy in lung cancer. However, these enzymes exert divergent effects on immune surveillance. Tripartite motif containing (TRIM)28 promotes the recruitment of myeloid-derived suppressor cells (MDSCs) and confers anti-PD-1 resistance. It catalyzes K63-linked ubiquitination of receptor-interacting serine/threonine kinase 1, thereby activating nuclear factor-κB (NF-κB) signaling (50). This process is dependent on its E3 activity. Additionally, the DNA-binding protein TRIM35 enhances immune surveillance in NSCLC. It mediates the K63-linked ubiquitination of lysine specific demethylase 1, which suppresses its demethylase activity. This inhibition subsequently leads to an upregulation of the transcription of the protein ER-Golgi intermediate compartment 1, stabilizes IFN-γ receptor 1 and activates IFN-γ signaling, ultimately leading to an increased expression of MHC-I (51). Similarly, FBW7 ubiquitinates PD-1 via K48-linked polyubiquitination, thereby targeting it for degradation and enhancing anti-PD-1 efficacy (52). This interaction is facilitated by cyclin-dependent kinase 1-mediated phosphorylation of PD-1 at the residue S261. Furthermore, tryptophan metabolism also engages in this cross-talk: Tryptophan or indoleamine 2,3-dioxygenase inhibitors have been shown to promote the tryptophanylation and activation of the E3, thyroid hormone receptor interactor 12 (TRIP12) at K1136. TRIP12 subsequently degrades nuclear factor of activated T cells 1 (NFATc1), which functions as a key transcription activator of PD-1(53). This pathway enhances CD8^+^ T-cell-mediated cytotoxicity and inhibits tumor growth.

The roles of DUBs in lung cancer immunity are context-dependent, as exemplified by the contrasting, yet complementary, functions of USP7 and USP12. Although both are cysteine proteases within the USP family, their inhibition leads to divergent outcomes in the tumor microenvironment, thereby highlighting the nuanced regulation of immunotherapy efficacy. The targeted inhibition of USP7 presents a dual therapeutic advantage; on one hand, it directly suppresses tumor growth through the activation of the p38 MAPK pathway, whereas, on the other hand, it concurrently effects an upregulation of PD-L1 expression (54). This seemingly paradoxical increase in PD-L1 serves to sensitize the tumor to anti-PD-1 antibodies, which thereby transforms the microenvironment into a more responsive state for combination immunotherapy.

Alternatively, USP12 acts as a guardian of antitumor immunity. Its knockdown exacerbates tumor progression through fostering a powerfully immunosuppressive niche. This occurs through enhanced macrophage recruitment, hypervascularization and T-cell inactivation. Mechanistically, a depletion of USP12 disrupts the stability of the protein phosphatase Mg^2+^/Mn^2+^ dependent 1B, which serves as a negative regulator of NF-κB signaling (55). The subsequent hyperactivation of NF-κB then drives the overexpression of pro-tumor chemokines, including CXCL1, CXCL8, CCL2 and CCL5, which promote the recruitment of tumor-associated macrophages and myeloid-derived suppressor cells (55). These chemokines, in turn, orchestrate the immunosuppressive landscape and drive resistance to immune checkpoint blockade (55). Therefore, a loss of USP12 function has the effect of crippling the responsiveness of tumors to anti-PD-1 treatment.

In summary, USP7 inhibition creates a favorable condition that may be combined with existing immunotherapies, whereas USP12 activity is essential for maintaining the capacity to respond to such treatments. This delineation underscores that a strategic targeting of the ubiquitin system requires a precise understanding of each component's unique role in balancing tumor suppression and immune activation.

### Ubiquitin-mediated modulation of oncogenic signaling and complementary checkpoints

Ubiquitin-dependent fine-tuning of the PI3K/AKT and MAPK cascades orchestrates the amplitude and duration of signaling that ultimately dictate the PD-1/PD-L1 checkpoint threshold in lung cancer. Within the PI3K-AKT axis, E3s such as WW domain-containing E3 ubiquitin protein ligase 2 promote Akt activation via mediating phosphatase and tensin homolog degradation. This consequently leads to an upregulation of PD-1 expression (56). On the other hand, PD-1 engagement itself attenuates Akt phosphorylation (57), thereby inducing metabolic quiescence in T cells (58). Similarly, ubiquitination has been shown to regulate multiple levels of the MAPK cascade. For example, Cbl-mediated ubiquitination of the epidermal growth factor receptor (EGFR) restricts upstream signaling, whereas the ubiquitin-dependent turnover of MAPK kinase/MAPK shapes downstream output (59). Collectively, these events lead to reduced ERK activity and lower PD-L1 expression (60). Therefore, dynamic ubiquitination and deubiquitination cycles not only modulate oncogenic signaling, but they also serve to calibrate the balance between T-cell exhaustion and reinvigoration, providing a mechanistic rationale for targeting ligases and DUBs to enhance immune checkpoint blockade.

DUBs markedly shape the immunosuppressive lung cancer microenvironment through controlling the stability of pivotal immune checkpoint molecules. Ubiquitin-specific peptidase 2 (USP2) directly deubiquitinates and stabilizes CD47, thereby stabilizing the protein and preserving its anti-phagocytic signal. Either genetic or pharmacological inhibition of USP2 has been shown to promote CD47 degradation, which thereby shifts macrophages toward a pro-inflammatory M1 phenotype, also enhancing CD8^+^ T-cell infiltration (61). In parallel, ubiquitin-specific peptidase 8 (USP8) circumvents the TNF receptor-associated factor 6-mediated K63-linked ubiquitination of PD-L1. Inhibiting USP8 directs PD-L1 toward K48-linked polyubiquitination and degradation, while simultaneously enhancing the NF-κB-dependent expression of MHC-I. Elevated MHC-I surface density increases the presentation of tumor-derived antigenic peptides to CD8^+^ T cells, thereby augmenting antigen presentation and priming cytotoxic T-cell responses (62). Consequently, the dual targeting of USP2 and USP8 disrupts two complementary ubiquitin-dependent immune barriers, namely phagocytic checkpoint and T-cell suppression, thereby effectively converting an immune-excluded tumor niche into an inflamed microenvironment that is susceptible to PD-1 checkpoint blockade.

### Dynamic equilibrium of the ubiquitin network in PD-1/PD-L1 immunotherapy

Notably, the fate of the immune checkpoint PD-L1 is not sealed at its production; rather, its fate is perpetually decided by a PTM ‘tug-of-war’ that is orchestrated within lung cancer cells. One the one hand, the E3s strive to pull PD-L1 toward its degradation, whereas, on the other hand, the DUBs counteract the activity of the E3s, and their function is to ensure the survival and continued activity of PD-L1. This dynamic equilibrium acts as a ‘master regulator’ of tumor immune evasion.

E3s modulate PD-L1 in various ways to cause its degradation, thereby exposing the tumor to immune attack. Ligases such as SPOP (activated by canagliflozin), FBXO22 and AIP4 in combination with MTSS1 directly bind to PD-L1. They decorate PD-L1 with ubiquitin chains, thereby marking it for proteasomal or lysosomal disposal. Other players, such as Cbl-b/c-Cbl and RNF182, however, take a more indirect approach. They do not target PD-L1 itself, but interrupt its production; Cbl-b/c-Cbl dampens the signaling pathways (such as the STAT3/AKT/ERK pathways) that drive PD-L1 transcription (63), whereas RNF182 downregulates a key transcription factor, p65, that is required for the activation of the PD-L1 gene (64).

Conversely, DUBs antagonize E3-mediated ubiquitination by removing ubiquitin chains to rescue PD-L1 from degradation. DUBs such as USP51, USP5 and OTUB2 act as notable defenders. They directly intercept PD-L1, and strip off its ubiquitin chains. A key tactical maneuver is employed by OTUB2, which interacts with PD-L1 during its processing in the endoplasmic reticulum (ER). This prevents its degradation before it even reaches the cell surface. Some DUBs, such as USP22, have been shown to provide a layered defense. USP22 not only directly deubiquitinates and stabilizes PD-L1, but also enhances the stability of CSN5, which itself functions as a PD-L1 stabilizer. This establishes a cooperative positive feedback loop that amplifies PD-L1 abundance (43).

The ultimate level of PD-L1 to be found on the tumor cell surface (and therefore, its ability to suppress T-cells) is the net result of this continuous ‘tug-of-war’. When E3s gain the upper hand, immunity is enhanced (51,52), but when DUBs dominate, the tumor persists. This refined understanding of the crosstalk moves us beyond a static list and reveals a dynamic signaling network. The most promising therapeutic strategies that are coming into prominence now aim to disrupt this balance. In terms of influencing this balance, clinicians can either inhibit the stabilizing DUBs (for example, with USP51 or OTUB2 inhibitors) or activate specific E3s. Through strategically intervening in this tug-of-war, it is possible to tip the scales in favor of the immune system and thereby overcome resistance to immunotherapy.

The success of anti-PD-1 therapy in lung cancer is therefore governed by a dynamic equilibrium in the ubiquitin system, where E3s and DUBs engage in a molecular tug-of-war to control immune recognition. E3s predominantly propel the system toward immune activation (65) through dismantling inhibitory checkpoints. FBW7 and TRIP12 have been shown to degrade both PD-1 and its transcriptional regulator, NFATc1. By contrast, TRIM35 potentiates T-cell surveillance by enhancing IFN-γ signaling. On the other hand, DUBs provide nuanced, context-dependent counterbalances to the action of the E3s. The contrasting roles of USP7 and USP12 exemplify this complexity. Inhibiting USP7 creates a synthetically vulnerable state wherein tumor cells become sensitized to immune checkpoint blockade: USP7 inhibition concurrently suppresses tumor growth through p38 MAPK activation and upregulates PD-L1 expression, thereby priming the tumor microenvironment for enhanced anti-PD-1 efficacy (54). By contrast, preserving USP12 is essential to prevent the collapse of the immune microenvironment into a state of profound immunosuppression (55).

Therefore, the ubiquitin system acts not merely as an on/off switch, but as a sophisticated signaling hub. Therapeutic success hinges on being able to strategically manipulate this hub. This strategic manipulation may be achieved either through reinforcing the ‘pro-immunity’ force of key E3s or by precisely inhibiting the ‘pro-resistance’ function of specific DUBs. Such interventions tip the scales in favor of sustained antitumor immunity (65).

## 3. LAG-3: An immune-checkpoint hub bridging T-cell exhaustion and tumor immune escape

The gene known as *LAG-3*, which was discovered in 1990 and was found to be a structural homologue of CD4, has been revealed to be expressed by various types of lymphocytes and non-lymphocyte lineage cells (66). LAG-3, an inhibitory receptor that is notably expressed in exhausted T cells, has been identified as a potential target for immunotherapy. LAG-3 has been shown to exert a regulatory function characterized by the suppression of cell proliferation, activation, effector function and homeostasis for both CD8 and CD4 T cells. Furthermore, this process disrupts the shared pathway of CD4 and CD8 activation, while concurrently regulating the activation and proliferation of T memory cells (67). As with PD-1, the constitutive expression of LAG-3 is often observed under conditions of T-cell exhaustion, and its expression is generally accepted as a hallmark of CD4 and CD8 T-cell exhaustion in response to repetitive antigenic stimulation in the context of cancer and chronic viral infections (68). In terms of the underlying mechanism, the expression of LAG-3 is stimulated either by TCRs or by cytokine stimulation (69). LAG-3 subsequently binds to TCR-CD3 complexes on the T-cell membrane, thereby exerting a negative regulatory effect on TCR signaling (53). This process ultimately leads to an arrest of cell proliferation and of cytokine secretion for the cells; in fact, LAG-3 has been demonstrated to physically interact with TCRs in CD8 and CD4 T cells following TCR engagement (69). This interaction results in downregulation of the TCR-dependent signaling cascade, with the subsequent suppression of T-cell responses (70).

MHC-II is widely regarded as the archetypal ligand of LAG-3, which interacts stably with the D1 domain. Following the binding of LAG-3 to MHC-II, CD4 T-cell activation becomes inhibited (71). Furthermore, it has been demonstrated that the binding of LAG-3 to MHC-II facilitates tumor evasion from apoptosis (72) and enhances the recruitment of tumor-specific CD4 T cells, while concomitantly reducing CD8 T-cell responses (73). Notably, the binding of galectin-3 (Gal-3) to LAG-3 is a prerequisite for the inhibition of CD8 T-cell cytotoxic function (74). The role of Gal-3 in anti-tumor immune responses is two-fold: First, it has been demonstrated that Gal-3 inhibits the activation of antigen-committed CD8 T cells; secondly, Gal-3 inhibits the expansion of plasmacytoid dendritic cells through LAG-3 expression in the tumor microenvironment (75). These effects, when considered collectively, form an antitumor immune response (75).

Fibrinogen-like protein 1 (FGL1), also referred to hepassocin, liver fibrinogen-related gene-1 or hepatocyte-derived fibrinogen-related protein, is a component of the fibrinogen-related protein family. It comprises two 34 kDa homodimers that are linked by disulfide bonds to form a 68-kDa protein (76), and contains two distinct domains: The N-terminal signal recognition peptide (helical domain, coiled-coil domain) and the C-terminal fibrinogen-like domain, which binds LAG-3 in the absence of an involvement of the membrane cross-over region (77,78). FGL1 is a novel ligand for LAG-3, in addition to its classical ligand, MHC-II. It forms a novel immune checkpoint pathway that is independent of PD-1/PD-L1, which results in T-cell exhaustion and subsequent dysfunction, as well as evasion of immune surveillance by tumor cells (79).

The expression of LAG-3 in T cells is widely regarded as a hallmark of aggressive tumor progression across a wide range of human tumors, especially in SCLC (79). Previous studies have demonstrated that tumors exhibiting immune evasion, primarily mediated by LAG-3, have a reduced sensitivity to PD-1 blockade (80,81). This provides a foundation for the potential future utilization of LAG-3 as a stratified biomarker in immunotherapy (69).

Emerging evidence has suggested that ubiquitin-mediated post-translational regulation notably governs both LAG-3 stability and function in lung cancer. The Cbl family, comprising c-Cbl, Cbl-b and Cbl-3, represents a class of RING-finger E3s that are characterized by a tyrosine kinase-binding ‘antenna’, an E2-docking RING ‘catalytic core’ and a C-terminal ‘signal relay’ domain composed of proline-rich and ubiquitin-associated/leucine zipper motifs (82,83). In NSCLC, these ligases function as gatekeepers of immune evasion. c-Cbl and Cbl-b mediate the redundant ubiquitination of LAG-3, which has the effect of trapping the receptor in a membrane-dissociated, signaling-silent state. This mechanism positions the LAG3-to-Cbl expression ratio as a readily applicable biomarker for predicting the response to anti-LAG3 therapy (84). Complementing this axis, FBXO38, an F-box component of the SKP1-cullin-F-box E3 ligase complex, acts as a metabolic rheostat in immune evasion. Through conjugating K48-linked ubiquitin chains to the immunosuppressive ligand FGL1, FBXO38 targets it for proteasomal degradation. This process not only suppresses interleukin (IL)-6-driven pro-tumor inflammation, but it also facilitates the infiltration of CD8^+^ T-cells into tumors. Clinically, a FBXO38-high/FGL1-low/IL-6-low expression signature identifies early-stage, immune-infiltrated NSCLC lesions, and this signature serves as an independent predictor of favorable outcomes following immune checkpoint blockade (85). Considered together, Cbl-mediated checkpoint desensitization and FBXO38-directed ligand clearance underscore a unifying biological principle: Specific E3s can be harnessed as ‘immune-tuning amplifiers’ that shift the ubiquitin equilibrium away from tumor-permissive states and toward tumor-restrictive immunity. These mechanisms offer not only quantifiable biomarkers for clinical analysis, but also rational combination strategies that may be employed to enhance the efficacy of PD-1/LAG3-targeted therapies.

## 4. B7-H4: An emerging immune checkpoint and the USP2a-driven axis

The co-inhibitory molecule B7-H4 is a notable constituent of the B7 family. It is a glycosylated molecule with a molecular mass of 50-80 kDa (86), and is a type-I transmembrane protein composed of 282 amino acids. As demonstrated in several studies, human B7-H4 mRNA is broadly expressed in numerous tissue types, including the placenta, liver, skeletal muscle, kidney, pancreas, prostate, testis, small intestine, stomach, spleen, lung, thymus, uterus, skin, lung, heart and brain (87). In NSCLC, elevated expression levels of B7-H4 are notably associated with lymph node metastasis and tumor-infiltrating lymphocytes (TILs) (88). In terms of the underlying mechanism, B7-H4 exerts its immunosuppressive effects through suppressing the activation, proliferation and clonal expansion of both CD4^+^ and CD8^+^ T lymphocytes. It simultaneously downregulates the secretion of key cytokines, including IL-2 and IFN-γ (89). This immunosuppressive activity is primarily mediated by the inhibition of TCR-mediated signaling cascades, ultimately resulting in T-cell dysfunction. Furthermore, B7-H4 facilitates tumor progression through activating diverse intracellular signaling pathways that enhance cancer cell proliferation, metastatic potential and resistance to programmed cell death (90).

In EGFR-mutant lung cancer, mutant EGFR signaling leads to an upregulation of the DUB USP2a. USP2a, in turn, stabilizes the immune checkpoint protein B7-H4 through cleaving its K48- and K63-linked ubiquitin chains, thereby shielding it from proteasomal degradation (91). This USP2a-mediated stabilization enhances the surface expression of B7-H4, thereby leading to suppressed T-cell activation and facilitated tumor immune evasion (91). The positive association observed between the protein levels of USP2a and B7-H4 in patient-derived specimens further validates this axis, and positions USP2a as a promising druggable target for restoring anti-tumor immunity.

## 5. Treatment strategies

### ICIs of lung cancer

ICIs perform a pivotal role in the management of different types of lung cancer, especially NSCLC. PD-1 inhibitors, such as nivolumab and pembrolizumab, are currently employed in clinical practice based on phase III randomized controlled trials (92,93), while PD-L1 inhibitors, such atezolizumab, have also demonstrated efficacy (94). Furthermore, for the first-line treatment of NSCLC, tremelimumab (Imjudo), a CTLA-4 inhibitor, has been used in combination with duvalumab (95). Drug resistance has gradually emerged with the widespread application of immunotherapy, although only a small proportion of patients with lung cancer respond to immunotherapy, and this in itself poses a new challenge in the treatment of lung cancer (14,96).

### Potential therapeutic approaches targeting ubiquitinases and DUBs

Berberine (BBR) is a naturally occurring isoquinoline quaternary alkaloid that is isolated from *Coptis chinensis*, a species of goldthread flowering plant native to China. It exhibits broad pharmacological properties, including anti-cancer, anti-microbial, anti-diabetic and anti-inflammatory activities (97). In terms of the underlying mechanism, BBR has been shown to enhance T-cell-mediated tumor cell death through downregulating the expression of PD-L1 in cancer cells. Specifically, BBR interacts with the glutamate residue E76 within CSN5, thereby inhibiting its DUB activity. This interaction promotes PD-L1 ubiquitination, and subsequent proteasomal degradation, which effectively disrupts the PD-1/PD-L1 immune checkpoint axis (97). Furthermore, BBR has been shown to impair the MPN domain of CSN5, which is critical for CSN5-mediated stabilization and deubiquitination of PD-L1(98). In addition to its direct effects on PD-L1, BBR also enhances anti-tumor immunity through promoting the infiltration and activation of tumor-specific T cells. It has also been shown to suppress the expansion and function of immunosuppressive cell populations, such as MDSCs and regulatory T cells (97).

P5091 is a selective USP7 inhibitor, which demonstrates potent immunomodulatory activity via reprogramming tumor-associated macrophages through activation of the p38 MAPK signaling pathway, which has the effect of promoting their polarization toward the M1 phenotype. This molecular mechanism enhances cytotoxic T lymphocyte-mediated tumor immune surveillance and ultimately suppresses tumor progression (54). Notably, USP7 inhibition causes an upregulation of PD-L1 expression within the tumor microenvironment, and this creates a synergistic therapeutic window when combined with PD-1 blockade immunotherapy (54).

Cullin 4A (Cul4A) is an 87-kDa protein, that belongs to a family of evolutionarily conserved cullin proteins and has been shown to be associated with the ubiquitin proteasome pathway (99). The virus protease inhibitor PIK-93 functions as a potent PD-L1 destabilizing agent through facilitating the formation of the CUL4A-E3 complex with PD-L1. This molecular interaction promotes the K48-linked polyubiquitination of PD-L1, thereby leading to its subsequent proteasomal degradation through the UPS (100). Administering PIK-93 has been shown to cause a notable downregulation of PD-L1 expression in M1-polarized macrophages, thereby augmenting their tumoricidal activity. In both syngeneic and human peripheral blood mononuclear cell-derived xenograft models, the combined administration of PIK-93 and PD-L1 blockade therapy has been demonstrated to potentiate T-cell-mediated antitumor immunity. This was evidenced both by an enhanced infiltration of TILs and a suppression of tumor progression (100). This combinatorial approach served to promote the establishment of an immunologically active tumor microenvironment, which led to a synergistic improvement of the therapeutic efficacy of immune checkpoint inhibition (100).

Metformin is a widely used anti-diabetic agent. Emerging research has demonstrated that it exhibits notable anti-tumor activity across multiple types of cancer, including lung, prostate and colorectal malignancies (101). Moreover, mechanistic studies have revealed that metformin-mediated stabilization of the adaptor protein, stimulator of interferon genes (STING), and the enhancement of T-cell cytotoxicity are both axis inhibition protein 1 (AXIN-1)-dependent. The genetic ablation of this scaffold protein led to an abrogation of these effects. In serine/threonine kinase 11-mutant lung cancer models, metformin was shown to synergize with the PD-1 blockade via suppressing RNF5-dependent K48-linked polyubiquitination, and the subsequent degradation of STING. This process was shown to require AXIN-1(102). Furthermore, metformin was found to potentiate tumor-specific T-cell responses *in vitro*, and to augment the therapeutic efficacy of PD-1 inhibitors in both preclinical models and experimental settings.

The antidiabetic drug canagliflozin has been shown to suppress tumor growth by reducing the expression of PD-L1 in an ‘on-target’ manner. In terms of the underlying mechanism, SGLT2 maintains PD-L1 stability during endocytic recycling through its direct interaction with it; however, canagliflozin was shown to have a two-fold effect, namely to disrupt the interaction between SGLT2 and PD-L1 and to promote the interaction between PD-L1 and the E3, SPOP (37).

**Figure 3:**
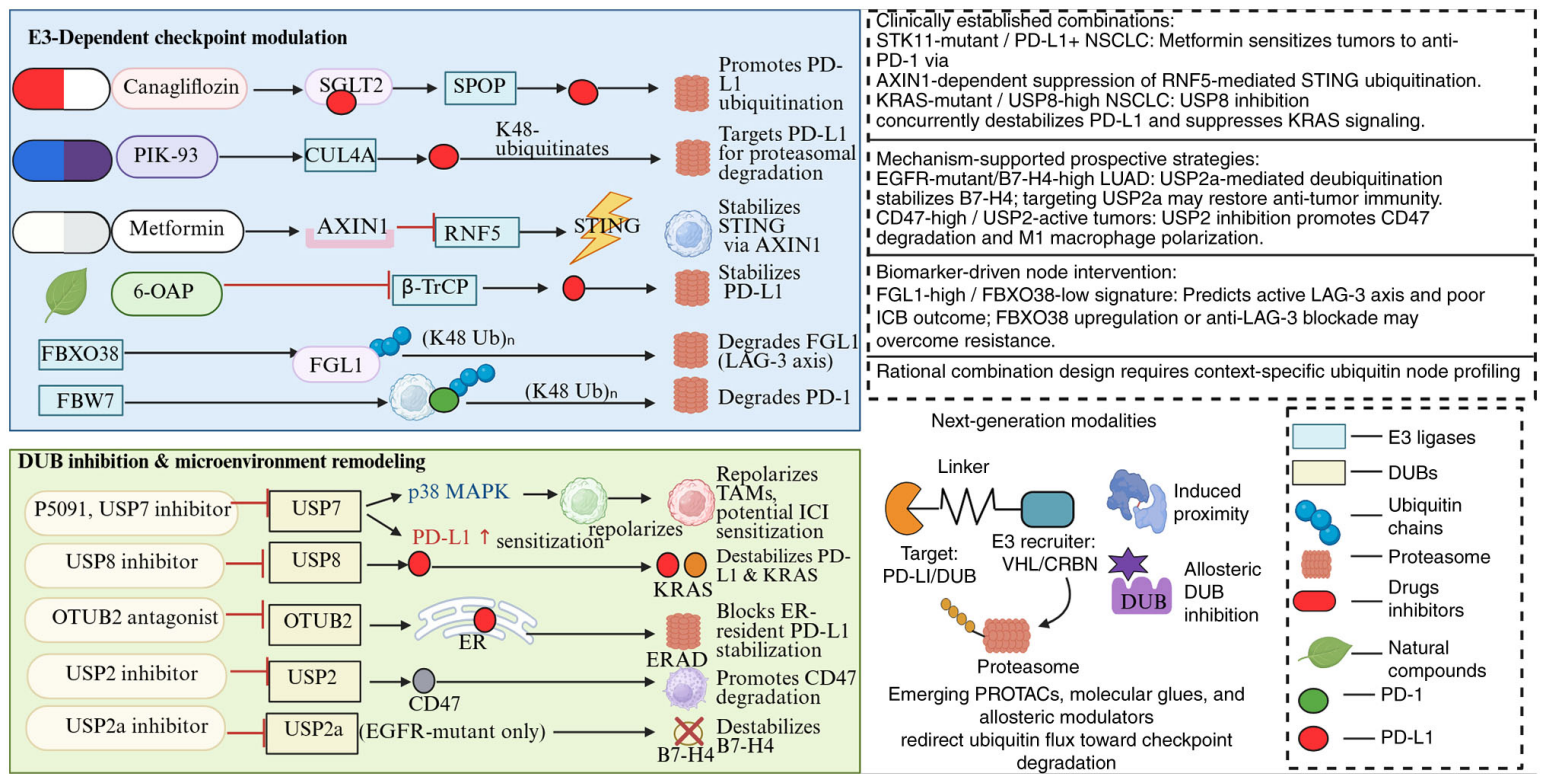
Therapeutic targeting of ubiquitin-modifying enzymes to enhance immune checkpoint blockade in lung cancer. The left panel illustrates E3 ligase activation strategies (canagliflozin/SPOP, PIK-93/CUL4A, metformin/AXIN1, 6-OAP/β-TrCP, FBXO38, FBW7) and DUB inhibition approaches (P5091/USP7, USP8, OTUB2, USP2, USP2a) that modulate immune checkpoint stability and tumor microenvironment remodeling. The right upper panel summarizes clinically established and prospective biomarker-driven combination strategies. The lower right panel depicts next-generation modalities, including PROTACs, molecular glues and allosteric modulators. PD-1, programmed cell death protein 1; PD-L1, programmed death-ligand 1; DUBs, deubiquitinating enzymes; SPOP, speckle-type POZ protein; FBXO22, F-box protein 22; FBW7, F-box and WD repeat domain-containing 7; RNF5, ring finger protein 5; USP, ubiquitin-specific peptidase; OTUB2, OTU and ubiquitin aldehyde-binding 2; ER, endoplasmic reticulum; SGLT2, sodium-glucose cotransporter-2; CUL4A, cullin 4A; AXIN1, axis inhibition protein 1; FGL1, fibrinogen-like protein 1; β-TrCP, β-transducin repeat-containing protein; STING, stimulator of interferon genes; LAG-3, lymphocyte-activating gene 3; TAMs, tumor-associated macrophages; ICI, immune checkpoint inhibitor; ERAD, ER-associated protein degradation; B7-H4, B7 homolog 4; STK11, serine/threonine kinase 11; NSCLC, non-small cell lung cancer; PROTAC, proteolysis-targeting chimera; VHL, Von Hippel-Lindau; CRBN, cereblon; EGFR, epidermal growth factor receptor; KRAS, Kirsten rat sarcoma viral oncogene homolog; ICB, immune checkpoint blockade.

PD-L1 also forms a complex with glycogen synthase kinase-3β (GSK-3β) and the E3 β-transducin repeat-containing protein (TrCP), leading to phosphorylation-dependent degradation of the UPS (103). The annual herb *Centipeda minima* (CM) and its active component 6-O-angeloylplenolin (6-OAP) were found to upregulate the expression of PD-L1 via inhibiting GSK-3β/β-TRCP-mediated ubiquitination and degradation, which led to further increases in CD8 T-cell infiltration (104). Therefore, mechanistically speaking, the induced expression of PD-L1 and the enhanced cytotoxicity of CD8 T cells underlie the beneficial effects of 6-OAP-rich CM in NSCLC. Preclinical studies have demonstrated that CM and 6-OAP significantly enhance ICI-induced tumor burden reduction and prolong overall survival in NSCLC-bearing mice when administered in combination with anti-PD-L1 antibody (104). These findings position CM as a promising candidate for combination with ICIs, and a phase I clinical trial (NCT05735028) is currently underway to evaluate its safety and efficacy in patients with lung cancer. As shown in Fig. 3, these diverse strategies converge on a unified therapeutic principle: Strategically tipping the ubiquitin equilibrium toward checkpoint degradation or microenvironment remodeling can overcome immunotherapy resistance in lung cancer (Table II and Fig. 3).

## 6. Discussion

E3s and DUBs perform a notable role in lung cancer pathogenesis through regulating the expression and signaling functionality of key immune checkpoint proteins, including PD-1, PD-L1, LAG-3 and B7-H4. Beyond directly modulating checkpoint activity through proteasomal degradation, these enzymes are also involved in the regulation of signaling pathways, including the EGFR, Wnt/β-catenin and NF-κB pathways (65), thereby indirectly shaping the immune landscape of the tumor microenvironment by influencing immune cell infiltration and the transcription of checkpoint molecules.

Notably, the functional outcomes of ubiquitin-modifying enzymes in lung cancer immunotherapy are not uniformly beneficial or detrimental. Rather, they are markedly context-dependent. For example, E3s such as SPOP, FBXO22 and FBW7 generally promote PD-L1 or PD-1 degradation to enhance anti-tumor immunity, DCUN1D1 paradoxically increases the expression of PD-L1 through FAK activation, thereby fostering immune evasion. Similarly, DUBs are not exclusively pro-tumorigenic. USP7 inhibition upregulates the expression of PD-L1, and yet it sensitizes tumors to anti-PD-1 therapy through remodeling macrophage polarization. By contrast, USP12 activity is essential for preserving an immunocompetent microenvironment. These observations both caution against simplistic categorization, and underscore the necessity of nuanced, context-specific therapeutic strategies.

In addition, ubiquitination and deubiquitination processes notably contribute to the efficacy of lung cancer treatments. They exhibit synergistic interactions with conventional therapies, such as chemotherapy and radiotherapy, while also enhancing the response to immunotherapy (105). For example, in chemotherapy, cisplatin has been shown to potentiate the antitumor effect of PD-L1 blockade through upregulating the expression of Ariadne RBR E3 ubiquitin protein ligase 1 (ARIH1) (106). ARIH1-mediated ubiquitination leads to the degradation of DNA-dependent protein kinase catalytic subunit. This, in turn, activates the STING pathway in tumor cells, thereby sensitizing them to immune checkpoint inhibition (106). In radiotherapy, ubiquitination and deubiquitination also modulate treatment outcomes. Radiation-induced DNA damage activates innate immune pathways such as the cyclic GMP-AMP synthase (cGAS)-STING pathway, thereby initiating a systemic T-cell response and abscopal effects. Notably, the activity of the cGAS-STING pathway is itself regulated by ubiquitination and deubiquitination (107). In the context of targeted therapy, these processes offer dual benefits, where they enhance immunotherapy efficacy, but also directly regulate oncogenic signaling. For example, the DUB USP8 promotes immune evasion and tumor progression via stabilizing both PD-L1 and Kirsten rat sarcoma viral oncogene homolog (KRAS) proteins. The inhibition of USP8 simultaneously suppresses KRAS signaling, and augments anti-PD-L1 efficacy, suggesting a promising strategy for treating KRAS-mutated lung cancer (62).

From a translational perspective, the most promising targets include SPOP activators (for example, canagliflozin) (37), USP7 inhibitors (for example, P5091) (40), USP8 inhibitors (62) and OTUB2 antagonists (106). These agents have demonstrated preclinical synergy with ICIs. Additionally, proteolysis-targeting chimeras (PROTACs) and allosteric modulators that selectively redirect ubiquitin flux toward checkpoint proteins have been shown to represent next-generation therapeutic modalities (108). Nevertheless, challenges impede clinical translation. First, the ubiquitin network exhibits substantial heterogeneity across tumor subtypes and individuals, and this is driven by tissue-specific enzyme expression and compensatory pathway redundancy. In addition, pharmacokinetic optimization, tumor-selective delivery and the management of on-target toxicities remain hurdles. Moreover, although several PROTACs and DUB inhibitors have entered phase I/II trials, the vast majority of ubiquitin-targeting drug candidates remain in the preclinical or early clinical stages (109-111), with limited clinical validation and efficacy data. This aspect highlights the urgent need to identify predictive biomarkers and to rationally design combination regimens.

## 7. Limitations

Several limitations should be acknowledged when interpreting the evidence presented in the current review. First, the majority of mechanistic studies to date have been conducted in specific cell lines or preclinical models (112), and these findings may not fully recapitulate the heterogeneity of human lung cancer *in vivo* (113). Secondly, the functional outcomes of ubiquitin modification are context-dependent; it has been demonstrated that the same E3 or DUB may exert divergent effects across different genetic backgrounds, disease stages or tumor microenvironments (114). For example, USP7 inhibition was shown to upregulate PD-L1 expression in certain contexts, and yet it suppresses tumor growth in others (115). This illustrates the complexity of predicting clinical outcomes. Thirdly, the depth of research varies across different checkpoints. PD-1/PD-L1 ubiquitin regulation is relatively well-characterized, whereas ubiquitin-associated studies for LAG-3, B7-H4, TIM-3 and TIGIT in lung cancer remain scarce. In addition, the majority of published translational studies have lacked validation in large clinical cohorts. The pharmacokinetic profiles, tumor selectivity and long-term toxicities of ubiquitin-targeting agents remain incompletely defined (108). Finally, compensatory mechanisms and pathway redundancy within the ubiquitin network may diminish the efficacy of single-target interventions (116). Considered collectively, these factors constrain the generalizability of current conclusions and underscore the need for cautious interpretation.

## 8. Conclusion

The present review systematically delineated the ubiquitin-driven regulation of immune checkpoints in lung cancer. It emphasized that the stability and function of PD-1, PD-L1, LAG-3 and B7-H4 are governed by a dynamic interplay between E3s and DUBs. Unlike prior reviews that have broadly surveyed ubiquitin mechanisms in cancer immunity (36), the present work has specifically integrated lung cancer pathobiology with checkpoint ubiquitination, highlighting context-dependent enzyme functions and bridging molecular mechanisms with emerging therapeutic strategies, such as PROTACs, DUB inhibitors and drug repurposing (108). It has been underscored that E3s and DUBs cannot be simplistically categorized as uniformly beneficial or detrimental to immunotherapy. Rather, their effects are dictated by substrate specificity, the cellular context and tumor microenvironmental cues. Looking forward, precision targeting of the ubiquitin system, guided by multi-omics profiling (117) and rational combination regimens, holds substantial promise for overcoming immunotherapy resistance. Nevertheless, challenges persist, including tumor heterogeneity, network redundancy and the need to identify clinically validated biomarkers. Future investigations should prioritize the mechanistic dissection of understudied checkpoints, including TIM-3 and TIGITS15). They should also develop selective enzymatic inhibitors and establish predictive frameworks to match specific ubiquitin targets with appropriate patient subpopulations. Over the course of the next 5 years, it is anticipated that first-in-class USP8 or OTUB2 inhibitors may enter early-phase clinical trials for patients with NSCLC who are resistant to PD-1 blockade.

## Figures and Tables

**Table I:** E3 ligases and DUBs associated with immune checkpoints in lung cancer.

Type	Name	Relevant immune checkpoints	Sites of ubiquitination or ubiquitin-linked chain	The role of immune therapy	(Refs.)
E3 ligase	TRIM28	PD-1	Lysine(s) within the RIPK1 kinase domain/K63-linked polyubiquitin chain	Inhibition	(50)
E3 ligase	FBW7	PD-1	Lys233 of PD-1/K48-linked polyubiquitin chain	Promotion	(52)
E3 ligase	TRIM35	PD-1	Lys422 of LSD1/K63-linked polyubiquitin chain	Promotion	(51)
E3 ligase	TRIP12	PD-1	Lysine 1136 of TRIP12/K48-linked polyubiquitin chain	Promotion	(53)
E3 ligase	SPOP	PD-L1	Lysine(s) within the cytoplasmic tail of PD-L1/K48-linked polyubiquitin chain	Promotion	(37)
E3 ligase	Cbl-b, c-Cbl	PD-L1	Lysines within STAT3, AKT, and/or ERK components/-	Promotion	(63)
E3 ligase	AIP4	PD-L1	Lys263 of PD-L1/mono-ubiquitination	Promotion	(39)
E3 ligase	FBXO22	PD-L1	Lysine(s) within the intracellular tail of PD-L1/lysine(s) within the intracellular tail of PD-L1	Promotion	(38)
E3 ligase	DCUN1D1	PD-L1	Indirect (no direct ubiquitination)	Inhibition	(41)
E3 ligase	RNF182	PD-L1	Lysine(s) within the RNF182 substrate p65/K48-linked polyubiquitin chain	Promotion	(64)
DUB	USP7	PD-1	Unidentified lysine(s) on p38 MAPK pathway component(s) or PD-L1 regulator(s)/-	Inhibition	(54)
DUB	USP12	PD-1	Unidentified lysine(s) within PPM1B/-	Promotion	(55)
DUB	USP51	PD-L1	Lys280 and Lys281 of PD-L1/removal of K48-linked polyubiquitin chains	Inhibition	(42)
DUB	USP22	PD-L1	-/removal of multiple non-K48 linkages-K6-, K11-, K27-, K29-, K33- and K63-linked ubiquitin chains	Inhibition	(43)
DUB	USP5	PD-L1	Indirect (no direct ubiquitination)	Inhibition	(48)
DUB	ATXN3	PD-L1	Lysine residues within IRF1, STAT3 and HIF-2α/removal of K48-linked polyubiquitin chains from IRF1, STAT3 and HIF-2α	Inhibition	(45)
DUB	UCHL1	PD-L1	Indirect (no direct ubiquitination)	Inhibition	(47)
DUB	OTUB2	PD-L1	-/removal of K48-linked polyubiquitin chains from PD-L1	Inhibition	(45)
E3 ligase	WWP2	PD-1	Lysine(s) within the PTEN phosphatase/K48-linked polyubiquitin chain	Inhibition	(56)
E3 ligase	CBL	PD-1	Lysine residues within EGFR, MAPKKK, MAPKK and MAPK isoforms/K48-linked polyubiquitin chain (proteasomal degradation) and K63-linked chain (non-proteolytic signaling)	Promotion	(59)
DUB	USP2	PD-L1	Lysine(s) within the CD47 cytoplasmic tail/removal of K48-linked polyubiquitin chains from CD47	Promotion	(61)
DUB	USP8	PD-L1	Lysine(s) within the intracellular tail of PD-L1/removal of K48-linked polyubiquitin chains from PD-L1	Promotion	(64)
E3 ligase	Cbl-b, c-Cbl	PD-1/LAG-3	Basic-residue-rich juxtamembrane region within the LAG3 cytoplasmic tail/K63-linked polyubiquitin chain	Promotion	(84)
E3 ligase	FBXO38	LAG-3	Lysine(s) within the FGL1 sequence/K48-linked polyubiquitin chain	Promotion	(85)
DUB	USP2a	B7-H4	Lysine(s) within the B7-H4 cytoplasmic tail/removal of both K48- and K63-linked polyubiquitin chains from B7-H4	Inhibition	(91)

TRIM28, Tripartite Motif-Containing Protein 28; RIPK1, Receptor-Interacting Serine/Threonine-Protein Kinase 1; FBW7, F-box and WD repeat domain-containing 7; PD-1, Programmed Cell Death Protein 1; PD-L1, Programmed Cell Death-Ligand 1; LSD1, Lysine-Specific Histone Demethylase 1; TRIM35, Tripartite Motif-Containing Protein 35; TRIP12, Thyroid Hormone Receptor Interactor 12; SPOP, Speckle-type POZ Protein; Cbl-b, Casitas B-lineage Lymphoma proto-oncogene b; c-Cbl, cellular Casitas B-lineage Lymphoma; STAT3, Signal Transducer and Activator of Transcription 3; AKT, AKT Serine/Threonine Kinase; ERK, Extracellular Signal-Regulated Kinase; AIP4, Atrophin-1 Interacting Protein 4; FBXO22, F-box protein 22; DCUN1D1, Defective in cullin neddylation 1 domain containing 1; RNF182, Ring Finger Protein 182; DUB, deubiquitinating enzyme; USP7, Ubiquitin-Specific Peptidase 7; MAPK, Mitogen-Activated Protein Kinase; USP12, Ubiquitin-Specific Peptidase 12; PPM1B, Protein Phosphatase, Mg²^+^/Mn²^+^ Dependent 1B; USP51, Ubiquitin-Specific Peptidase 51; USP22, Ubiquitin-Specific Peptidase 22; USP5, Ubiquitin-Specific Peptidase 5; ATXN3, Ataxin-3; IRF1, Interferon Regulatory Factor 1; STAT3, Signal Transducer and Activator of Transcription 3; HIF-2α, Hypoxia-Inducible Factor 2 Alpha; UCHL1, Ubiquitin C-terminal Hydrolase L1; OTUB2, OTU Deubiquitinase, Ubiquitin Aldehyde Binding 2; WWP2, WW Domain Containing E3 Ubiquitin Protein Ligase 2; PTEN, Phosphatase and Tensin Homolog; MAPKKK, Mitogen-Activated Protein Kinase Kinase Kinase; MAPKK, Mitogen-Activated Protein Kinase Kinase; USP2, Ubiquitin-Specific Peptidase 2; USP8, Ubiquitin-Specific Peptidase 8; LAG-3, Lymphocyte-Activation Gene 3; FBXO38, F-box protein 38; FGL1, Fibrinogen-Like Protein 1; USP2a, Ubiquitin Specific Peptidase 2 isoform a; B7-H4, B7 Homolog 4.

**Table II:**
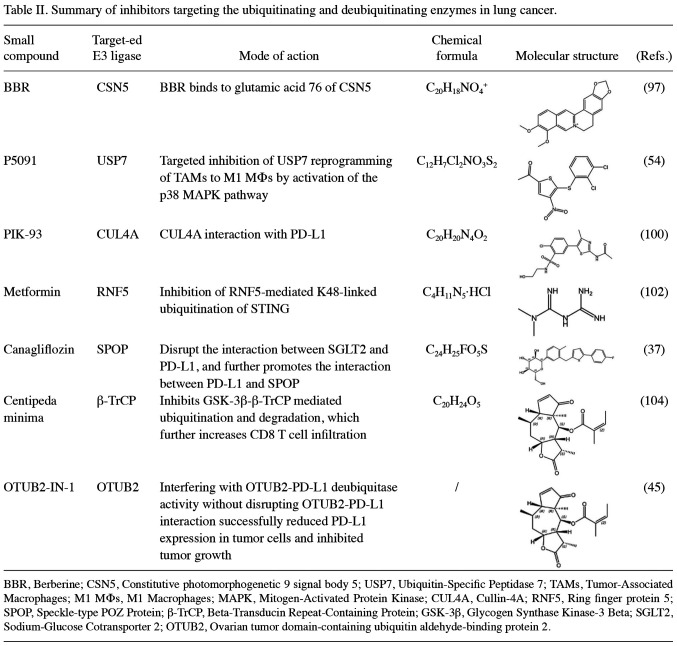
Summary of inhibitors targeting the ubiquitinating and deubiquitinating enzymes in lung cancer.

## Data Availability

Not applicable.
